# Insulin Resistance and Type 2 Diabetes in Asymptomatic Obstructive Sleep Apnea: Results of the PROOF Cohort Study After 7 Years of Follow-Up

**DOI:** 10.3389/fphys.2021.650758

**Published:** 2021-07-28

**Authors:** Laurine Vacelet, David Hupin, Vincent Pichot, Sébastien Celle, Isabelle Court-Fortune, Thierry Thomas, Arnauld Garcin, Jean-Claude Barthélémy, David Gozal, Frédéric Roche

**Affiliations:** ^1^Service de Physiologie Clinique et de l’Exercice, CHU Saint Etienne, Saint Etienne Cedex, France; ^2^Sainbiose DVH U1059 Inserm, Faculté de Médecine J Lisfranc, Université Jean Monnet, Saint Etienne Cedex, France; ^3^Service de Pneumologie, CHU Saint Etienne, Saint Etienne Cedex, France; ^4^Service de Rhumatologie, CHU Saint Etienne, Saint Etienne Cedex, France; ^5^URCIP, CHU Saint Etienne, Saint Etienne Cedex, France; ^6^Department of Child Health, MU Women’s and Children’s Hospital, Columbia, MO, United States

**Keywords:** obstructive sleep apnea, elderly, T2DM, HOMA–IR, cohort study, aging

## Abstract

The aim of the study was to assess potential associations between obstructive sleep apnea (OSA) and the occurrence of diabetes mellitus and insulin resistance in the elderly. Nondiabetic volunteers (*n* = 549) with undiagnosed or untreated asymptomatic OSA (66.2+/−1 years at the inclusion) were evaluated as an ancillary study of the PROOF cohort study (*n* = 1,011). After 7 years follow-up, 494 subjects underwent assessment of fasting insulin and glucose levels. OSA was defined by an apnea-hypopnea index (AHI) of ≥15/h using polygraphy. Diabetes mellitus was defined by a fasting glucose ≥ 1.26 g/L and/or when requiring pharmacological treatment, while insulin resistance corresponded to HOMA-IR ≥ 2. Asymptomatic OSA subjects (men or women) did not display increased risk of incident diabetes (2.8 vs. 3.9%, *p* = 0.51). However, there was a greater frequency of insulin resistance in subjects with severe OSA (AHI > 30) [OR 2.21; 95% CI (1.22–4.02); *p* = 0.009]. Furthermore, multiple logistic regression showed that triglycerides levels [OR 1.61; 95% CI (1.10–2.36); *p* = 0.01] and fasting glycaemia [OR 4.69; 95% CI (1.12–192.78); *p* = 0.04], but not AHI or oxyhemoglobin desaturation index were independently associated with higher rate of insulin resistance. The deleterious metabolic effect of asymptomatic OSA in the population may be indirectly mediated via perturbations in lipids, and is particularly likely to become manifest in severe apneic subjects with higher glycemic levels.

## Introduction

Obstructive sleep apnea (OSA) and type 2 diabetes (T2DM), are two chronic conditions that are increasingly prevalent worldwide, particularly affecting the elderly population. Studies focusing on the prevalence of OSA have generally shown that it is an age-related disorder (Mehra et al., 2007), whereby OSA prevalence increases with advancing age, and reaches a plateau around 65 years of age and thereafter (Young et al., 2002). Similarly, epidemiological studies have reported a high prevalence of T2DM between 65 and 74 years of age (estimated at 18%), but followed by a decrease in those individual older than 75 years (estimated at 5.5%) (Narayan et al., 2006). Both OSA and T2DM exhibit increased risk of several cardiovascular disease (CVD) and complications, albeit via different pathophysiological mechanisms. OSA is defined by the occurrence of at least five events per hour of partial (hypopnea) or complete (apnea) collapse of the upper airways and is usually manifest as daytime hypersomnolence or at least two of the following signs: snoring, nocturia, multiple nighttime arousals, nocturnal suffocation, nonrestorative sleep, daytime fatigue, and cognitive dysfunction. When untreated, OSA is associated with an increased incidence of hypertension, myocardial infarction, heart failure, ischemic stroke, and cardiac arrhythmias, including atrial fibrillation (Young et al., 1997; Gottlieb et al., 2010; Redline et al., 2010). Similarly, T2DM can be complicated by micro- and/or macrovascular dysfunction, including stroke, lower limb ischemia, and myocardial infarction. The cardiovascular complications of both T2DM and OSA are secondary to accelerated atherosclerosis and endothelial dysfunction, which are promoted by chronic hyperglycemia, systemic inflammation, and oxidative stress. In OSA patients already manifesting high risk CVD risk, T2DM generates incremental CVD risk, hence the importance of screening for both conditions systematically, and particularly during their early stages. In fact, each of these two pathological entities may be a consequence of the other, and their cross-prevalence is therefore very high with the prevalence of OSA in type 2 diabetics reaching 18%, and conversely, 15–30% of OSA patients suffering from T2DM. Both diseases have common risk factors such as age, male gender, high body mass index (BMI), and genetic predisposition, with likely common underlying pathophysiological processes accounting for this link. Several reviews have been carried out and focused on the reciprocal interactions between OSA syndrome and type 2 diabetes (Pamidi and Tasali, 2012).

Obstructive sleep apnea is characterized by chronic intermittent hypoxia, EEG and autonomic sleep fragmentation. In response to intermittent hypoxemia and recurrent arousals or micro-arousals, a set of deleterious pathophysiological events including sympathetic nervous system activation, decreased secretion or resistance to the satiety hormone leptin, increased oxidative stress (Li et al., 2018), sarcoplasmic reticular stress (Yi et al., 2017), impairments in corticotropic axis function, and release of inflammatory adipocytokines may impair glucose homeostasis, leading to insulin resistance and glucose intolerance (Moon et al., 2015). Animal models and human studies in healthy volunteers have confirmed the role of chronic intermittent hypoxia in altering glucose metabolism (Louis and Punjabi, 2009; Rafacho et al., 2013; Shin et al., 2014; Newhouse et al., 2017). Symptomatic OSA has clearly emerged in adults as an independent risk factor for insulin resistance and onset of T2DM (Anothaisintawee et al., 2016). Clinical studies suggest that insulin resistance assessed by the homeostastic model assessment (HOMA-IR) and glucose intolerance are positively associated with OSA, independent of the degree of obesity (Archontogeorgis et al., 2017). Two large recent prospective cohort studies, one from Canada and another from patients recruited in the Atherosclerosis Risk in Communities Study (ARIC) and/or in the Sleep Heart Health Study (SHHS), have shown an increase in T2DM prevalence that is related to OSA severity. Few comparable data are available, however, among older patients with OSA.

The main objective of the present study was to evaluate the impact of unrecognized OSA in a nondiabetic community population on the occurrence of T2DM or the rate of insulin resistance in subjects over 65 years of age after a period of 7 years. Secondary objectives were to assess whether autonomic sleep fragmentation and/or the oxyhemoglobin desaturation index (ODI) play a role in glucose metabolism dysregulation in this context.

## Materials and Methods

### Population

The study sample was selected from the PROOF cohort study. The PROOF study (PROgnostic indicator OF cardiovascular and cerebrovascular events) is a prospective observational cohort study of 1,011 volunteers, (all 65 years of age at baseline) which was designed to evaluate the prognostic value of autonomic nervous system (ANS) activity changes and their role in cardiovascular and cerebrovascular morbidity and mortality. The population was recruited consecutively from the electoral list of the city of Saint-Etienne, France, from 2000 to 2003.

Out of 3,983 eligible original participants, 11% refused to participate and 67% did not answer. The population therefore included 1,011 participants. In this study, exclusion criteria included a history of stroke, myocardial infarction, heart failure, cardiac pacemaker, insulin-dependent diabetes mellitus (T1D), atrial fibrillation or antiarrhythmic therapy, and severe diseases with a life expectancy of <5 years.

An additional sub study, the SYNAPSE study (SYsteme Nerveux Autonome–Physiologie–Sommeil–Epidemiologie), aimed to assess the association between sleep-disordered breathing, and cardiovascular, and cerebrovascular morbidity and was proposed as an add-on study from 2003 to 2006 to all participants included in the PROOF study. Inclusion criteria in the SYNAPSE study were the absence of previous stroke, myocardial infarction and atrial fibrillation, no prior diagnosis or treatment for OSA, willingness to perform an ambulatory overnight polygraphic recording. Subjects undergo 24-h ambulatory blood pressure and 24-h ECG Holter monitoring, and a blood draw. For the present glucose metabolic sub-study, subjects who were treated (after the first polygraphic assessment) for OSA (either by continuous positive pressure ventilation via a nasal mask or by mandibular advancement device), as well as those who had T2MD, i.e., those with fasting blood glucose ≥ 1.26 g/L and/or those taking one or more antidiabetic treatments (oral hypoglycemic agents or insulin therapy) were excluded.

**FIGURE 1:**
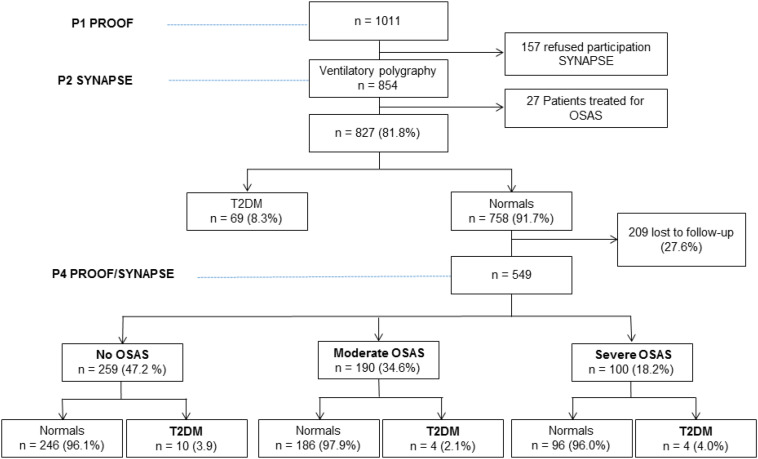
Flowchart of the study.

758 met the initial specific selection criteria, and 408 (53.8%) were diagnosed as suffering from OSA. Of these, 209 subjects were lost to follow-up or refused the clinical and/or biological assessment 7 years later (Figure 1).

Thus, the present cohort consisted of 549 subjects (Figure 1), with a majority being women [average age of 66.2 ± 0.9 (63–70) years].

All subjects were evaluated under standardized conditions as described previously (Barthélémy et al., 2007). The Advisory Ethics Committee for the Protection of Individuals in Medical Research (CPPRB Sud-Est 1) and Hospital Clinical Research Program in 1998 and 2001 approved this protocol (CHU Saint Etienne, France). All volunteers gave their written informed consent to participate. The Conseil National Informatique et Liberté (CNIL) stipulated how to save the data of this study. The protocols of the PROOF and SYNAPSE studies are available at clinicaltrials.gov (Clinical Trial Gov: NCT 00759304 and NCT 00766584).

### Clinical and Anthropometric Evaluation

All subjects underwent a clinical assessment, including a questionnaire on demographic characteristics, their medical history and their medications. The evaluation focused primarily on cardiovascular, cerebral, respiratory disorders, hypertension, diabetes, and psychiatric conditions. Treatments listed were antihypertensives, antidiabetics (oral and insulin therapy), hypnotics, and anxiolytics at inclusion and in the last examination follow-up of the cohort (Proof 4 examination). Anthropometric characteristics including gender, age, BMI, neck circumference, 24-h blood pressure, and total body composition measured by dual-energy X-ray absorptiometry (DXA) were obtained for all subjects. Subjects were defined as normotensive if they had no history of hypertension, did not take antihypertensives, and if the 24-h ambulatory blood pressure measurement did not find systolic blood pressure (SBP) > 135 mmHg and diastolic blood pressure (DBP) > 85 mmHg. All patients had an assessment of daytime sleepiness using a French version of the Epworth scale, with daytime hypersomnolence defined as >9/24 (Billiard et al., 1996). Respiratory sleep study was systematically assessed at each follow-up period regardless of the presence or absence of sleep respiratory disorders (known or discovered on this occasion).

### Study of Sleep Apnea

Unattended night respiratory polygraphic recordings were performed at home for all subjects using a dedicated recording system (HypnoPTT, Tyco Healthcare, Puritan Bennett). The system included a nasal pressure cannula, piezoelectric chest, and abdominal straps, a pulse oximeter to quantify oxygen saturation (SpO_2_), an electrocardiogram with a photoplethysmographic sensor to continuously measure pulse transit time and R–R interval, a microphone (for snoring quantification), and a positional sensor. Software was used for downloading and analyzing the tracings. A recording time ≥5 h was required. The evaluation was repeated a second night if the subjective latency of sleep was greater than 2 h the first night, if the recording lasted <5 h, or if the respiratory measures were missing.

All recordings were visually validated and manually scored for respiratory events and SpO_2_. Hypopnea was defined as a reduction in airflow ≥ 50% from baseline for at least 10 s and associated with oxygen desaturation ≥ 3%. Apnea was defined as an absence of airflow through the nasal cannula for ≥ 10 s. The absence of chest movements during apnea defined the event as central, whereas a gradual increase in chest wall movement and pulse transit time defined the event as obstructive. To minimize potential overestimation of sleep duration, subjects completed a sleep diary to set bedtime (lights off) and wake up time (lights on). The apnea-hypopnea index (AHI) was established as the ratio of the number of apneas and hypopneas per hour to the sleep time.

The severity of OSA was expressed in terms of AHI. According to recent data in the elderly, an AHI ≥ 15 events/h with at least 85% of events considered obstructive was considered as a diagnosis of OSA. Apneic patients were classified as mild or non-apneic subjects (AHI < 15/h), moderate OSA (15 ≤ AHI < 30/h), and severe OSA (AHI ≥ 30/h) (No Author, 1999; Pavlova et al., 2008). The parameters of nocturnal hypoxemia were as follows: mean SpO_2_, percentage of recording time spent with SpO_2_ < 90%, minimum SpO_2_ value recorded during sleep, and oxygen desaturation index (ODI) defined as the number of episodes of oxyhemoglobin desaturation ≥ 3% per hour based on total sleep time. An ODI ≥ 15/h was considered abnormal. Sleep fragmentation was defined by an index of autonomic micro-arousals (index of autonomic awakenings) ≥ 30/h using the pulse wave transit time method.

Pulse transit time (PTT) was calculated as the time interval between the electrocardiographic R wave and a point on the pulse waveform (detected by a plethysmographic finger probe) that was 50% of the height of ascent of the pulse wave. The electrocardiogram and pulse were sampled at 500 Hz. PTT is typically about 250 ms and is measured to an accuracy of 2 ms. PTT values available with every heart beat were oversampled at 5 Hz. The PTT was continuously monitored and an autonomic awakening index was obtained from the PTT signal, and was broken down into total, respiratory, and non-respiratory autonomic activations. The scoring of autonomic events was obtained using the manufacturer’s analysis software. Autonomic sleep fragmentation was defined by an index of autonomic sleep-related micro-arousals (ASR) ≥ 30/h using the pulse wave transit time method. The methodology of this technique was provided in great detail in a previous article describing the PROOF study (Barthélémy et al., 2007).

### Data Monitoring

Three 2-year interval examinations were initially scheduled for the 7 years of follow-up (2000–2007). Participants received an invitation by mail for each evaluation. Missing information was obtained from hospital records and questionnaires sent to the attending physician. After the first polygraphic evaluation, the results were sent to their primary care physician, who decided whether to initiate or not continuous positive airway pressure therapy or treatment with a mandibular advance device. Blood samples were taken by a fasting technician or nurse in the laboratory in the morning within a week of the respiratory polygraphic study. Presence of T2DM and of insulin resistance were assessed in the fourth assessment. T2DM was identified by a diagnostic test defined by fasting glucose ≥ 1.26 g/L and/or by antidiabetic treatment (oral hypoglycemic agents, insulin therapy; American Diabetes Association, 2010). Insulin resistance was defined at the same time-period by calculation of a HOMA-IR ≥ 2. This threshold of more than 2 was chosen because it seems in a prospective study of the general population the most accurate predictor of the occurrence of type 2 diabetes mellitus (Lee et al., 2016). HOMA-IR index calculation was carried out with the following formula: HOMA-IR = (fasting glucose mmol/L × fasting insulinemia pmol/L)/22.4. Insulinemia was measured by the MILLIPLEX^R^ MAP kit technique. Fasting glycaemia, serum lipid levels, including triglycerides and total high-density lipoprotein and calculated low-density lipoprotein cholesterol, were assessed using validated methodologies (Roche Diagnostic GmbH, Mannheim, Germany).

### Statistical Analyses

Statistical analysis was performed using Statview 5.1 software. The results were expressed in terms of absolute or percentage values, and adjusted odds ratios with corresponding 95% confidence intervals (CIs). A value of *p* < 0.05 was considered statistically significant. The use of one-factor ANOVA analysis for continuous variables and of contingency tables compared “T2DM” and “nondiabetic” groups as well as “insulin resistant” and “non-insulin resistant” groups. Logistic regression analysis assessed the role of clinical, anthropometric, biological, and polygraphic features at baseline that may increase the rate of T2DM and insulin resistance. We built and compared a series of models. Model 1 included gender and BMI ≥ 30 kg/m^2^. Model 2 additionally adjusted for whole body densitometry (fat mass) data. Finally, Model 3 also included fasting glucose and triglycerides in the prediction of presence of T2DM or insulin resistance.

## Results

### Population Characteristics at the Inclusion

Polygraphic assessments revealed the presence of moderate OSA in 34.5%, and 100 subjects met the criteria for severe OSA (18.2%) (Table 1). Of note, the severe OSA group was predominantly male (60%). Anthropometric characteristics (weight, BMI, hip circumference, and waist circumference) increased as AHI severity increased. The subjects were asymptomatic with an average Epworth scale score of 5.6 ± 3.6 (with a score of >11 considered as indicative of excessive daytime sleepiness). Fasting blood glucose and triglyceride levels in severe OSA patients were significantly higher than in the non-apneic group. Ambulatory SBP and DBP (mean 24-h) were also higher in the moderate and severe OSA groups. Approximately one-third of the subjects took at least one antihypertensive medication at the initial assessment (P1) of the study. DXA parameters were similar between groups with a mean whole body fat composition of 31.7 ± 8.3% and a trunk fat component of 32.3 ± 8.3%.

**TABLE 1:** Characteristics of the cohort upon initiation of the study (P1 PROOF).

OSA severity	Normal	Moderate	Severe	*P*-value
		
AHI/h *n* (%)	<15 *n* = 259 (47.2)	15.0–29.9 *n* = 190 (34.6)	≥30.0 *n* = 100 (18.2)	
Age (years ± SD)	66.1 (0.8)	66.1 (0.9)	66.2 (1.0)	NS
Gender, *n* (%)				
Females	184 (71)	110 (57.9)	40 (40)	<0.001
Males	75 (29)	80 (42.1)	60 (60)	
BMI kg/m^2^ (±SD)	24.2 (3.8)	25.7 (3.9)	26.8 (3.9)	< 0.0001
Waist circumference (cm) (±SD)	97.2 (9.0)	98.1 (9.3)	101.1 (7.5)	<0.0001
Hip circumference (cm) (±SD)	82.8 (10)	85.0 (11.7)	91.2 (9.9)	<0.0001
Hip/waist (±SD)	0.85 (0.06)	0.87 (0.10)	0.91 (0.09)	<0.0001
Epworth scale score (±SD)	5.0 (3.8)	5.7 (3.6)	6.4 (3.4)	0.001
Fasting glucose g/L (±SD)	0.97 (0.1)	0.97 (0.1)	0.99 (0.1)	0.01
Cholesterol g/L (±SD)	2.34 (0.42)	2.34 (0.37)	2.35 (0.52)	NS
LDLc g/L (±SD)	1.55 (0.38)	1.53 (0.35)	1.56 (0.45)	NS
HDLc g/L (±SD)	1.84 (0.41)	1.83 (0.44)	1.75 (0.58)	NS
Triglycerides g/L (±SD)	1.38 (0.68)	1.37 (0.70)	1.59 (0.93)	0.001
24-h Systolic BP mmHg (±SD)	115 (14)	119 (14)	122 (17)	0.001
24-h Diastolic BP mmHg (±SD)	73 (8)	75 (9)	77 (10)	0.001
Antihypertensive medication (%)	91 (35.1)	85 (44.7)	51 (51)	<0.01
Head fat mass* % (±SD)	20.3 (1.8)	20.5 (1.7)	20.9 (1.8)	NS
Trunk fat mass* (%) (±SD)	31.9 (8.9)	32.5 (9.5)	33.3 (9.8)	NS
Whole body fat mass* (%) (±SD)	32.2 (8.5)	31.5 (9.5)	31.1 (9.5)	NS

BMI, body mass index; HDLc, high density lipoprotrein cholesterol; LDLc, low density lipoprotein cholesterol; BP, blood pressure. *Body fat mass was measured using dual-energy X-ray absorptiometry.

### Incidence of Diabetes

Of the 549 subjects free of T2DM at entry and evaluated at the fourth visit, we found a 4% incidence of T2DM in the severe OSA group and only 2.1% in the moderate OSA group when compared to 3.9% (i.e., 10 subjects) in the non-apneic control group (*p* = 0.51).

Univariate analysis showed that waist circumference [OR 1.05, 95% CI (1.004–1.10), *p* = 0.03], trunk fat percentage [OR 1.72, 95% CI (1.11–2.67), *p* = 0.02] and neck/head fat content percentage [OR 1.07, 95% CI (1.01–1.15), *p* = 0.047] increased the risk of T2DM occurrence at 7 years. Furthermore, presence of a pre-diabetic state (defined as a blood glucose level between 1.10 and 1.26 g/L) increased the risk of developing T2DM at 7 years by 6.7 fold [OR 6.7, 95% CI (2.48–18.4), *p* = 0.0002].

**FIGURE 2:**
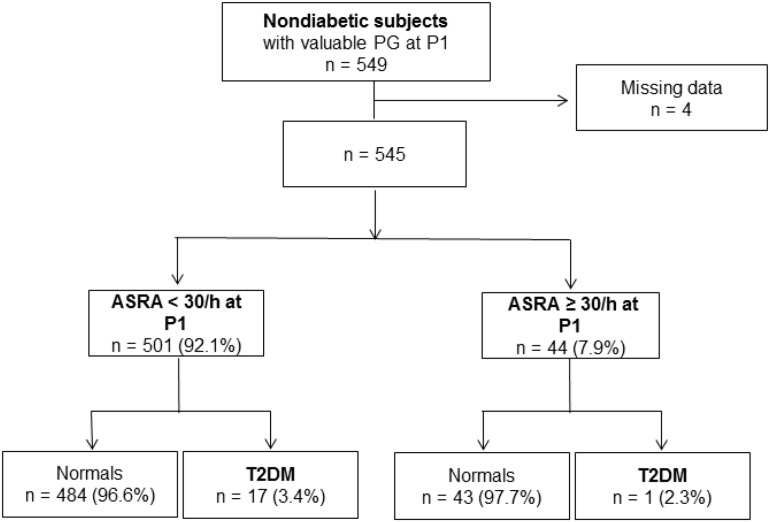
Autonomic sleep fragmentation index at the inclusion and its impact on diabetes incidence after 7 years.

Follow-up analysis assessed potential associations between sleep fragmentation and hypoxic indices and the incidence of T2DM. The presence of respiratory-related autonomic sleep fragmentation (micro-arousal autonomic index > 30/h) was not associated with increased incidence of diabetics was found [2.27 vs. 3.3% in normal; OR 1.51, 95% CI (0.20–11.63); *p* = 0.67; Figure 2].

**FIGURE 3:**
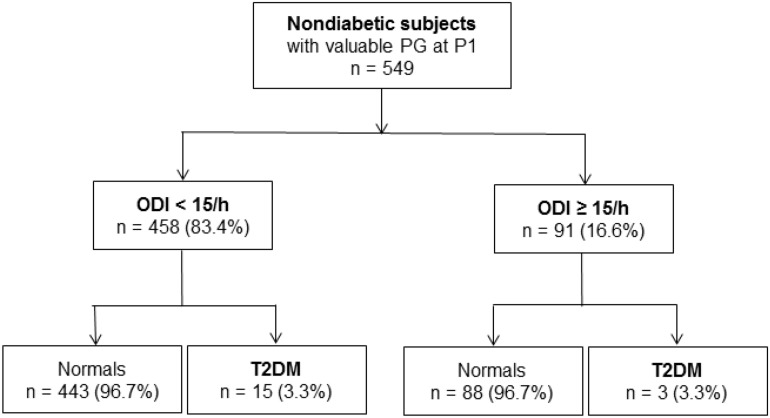
Hpoxemic load (oxyhemoglobin desaturation index) at the inclusion and its impact on diabetes incidence after 7 years.

The presence of chronic intermittent hypoxic stress defined as ODI > 15/h did not increase the incidence of T2DM (3.29% in the “ODI + group” compared to 3.27% in normal; OR at 0.99 [95% CI (0.28–3.50); *p* = 0.99; Figure 3].

### Rate of Insulin Resistance at 7 Years

There were 493 subjects with a validated plasma insulin level as well as fasting blood glucose concentration obtained during the 7-years follow-up visit. We should remark that due to technical issues with the insulin assays, insulin levels were not available in 56 subjects.

**FIGURE 4:**
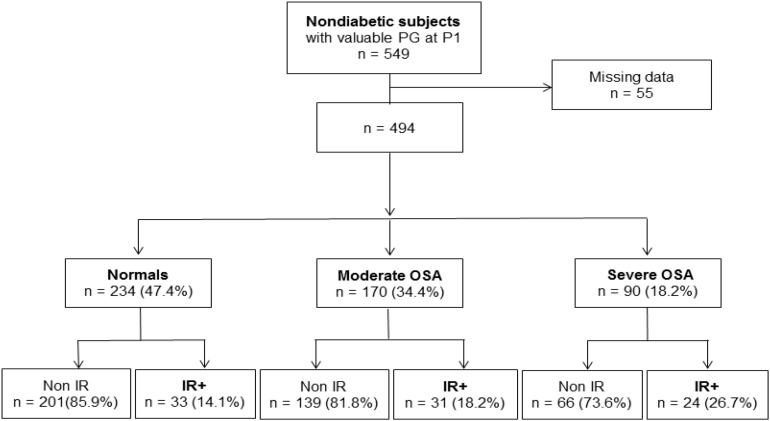
Obstructive sleep apnea severity at the inclusion and its impact on insulin resistance evaluated after 7 years.

We found that 26.7% (24 subjects) exhibited evidence of insulin resistance among the subjects with severe OSA, while 18.2% (31 subjects) had elevated HOMA-IR values in moderate OSA vs. 14.1% (32 subjects) in controls, thereby indicating a statistically significant effect of sleep apnea severity on the presence of insulin resistance (*p* < 0.03; Figure 4).

However, HOMA-IR values were not significantly different between OSA groups regardless of their obesity “status” (Table 2).

**TABLE 2:** HOMA-IR index according OSA severity as well as obesity or not.

Variables	All subjects	AHI < 15/h		15 ≤ AHI < 30/h		AHI ≥ 30/h		*p*-value
BMI m^2^/kg (±SD)	25.14 (3.72)	<30	≥30	<30	≥30	<30	≥30	
*n*	493	222	11	155	15	76	14	
HOMA-IR (±SD)	1.37 (1.77)	1.26 (1.77)	1.67 (0.79)	1.31 (1.72)	2.46 (3.06)	1.41 (1.41)	2.29 (2.40)	0.52

*BMI, body mass index; HOMA-IR, homeostasis model assessment of insulin resistance.*

In the subgroup analysis, an association was observed between the severity of OSA and the risk of insulin resistance. Severe apneic patients had an increased risk of suffering insulin resistance of 2.21 [OR 2.21; 95% CI (1.22–4.02); *p* = 0.009] with no effect being apparent among moderate OSA subjects [OR 1.36; 95% CI (0.80–2.32); *p* = 0.26]. However, ODI > 15/h and autonomic sleep-related micro-arousal index > 30/h were not associated with increased risk of insulin resistance [respectively, OR 1.23; 95% CI (0.68–2.23); *p* = 0.49 and OR 1.52; 95% CI (0.52–2.69); *p* = 0.67].

### Univariate Analysis

In the univariate analysis, significant predictors of insulin resistance included BMI of 30 kg/m^2^, fasting blood glucose (continuous variable) and the presence of trunk fat mass above the 90th percentile of the population {OR 3.13 [95% CI (1.58–6.22); *p* = 0.001], 26.36 [95% CI (2.64–263.55); *p* = 0.005}, and 3.38 [95% CI (1.71–66.6); *p* = 0.005, respectively; Table 3]. Other statistically significant parameters were BMI (continuous variable) with an OR at 1.04 [95% CI (1.02–1.06); *p* = 0.0001], hip circumference with OR at 1.03 [95% CI (1.02–1.06); *p* = 0.0001], waist circumference OR at 1.05 [95% CI (1.03–1.08); *p* = 0.0001], triglycerides level OR at 1.83 [95% CI (1,3–2.56); *p* = 0.0005], and fat mass of the trunk with OR 1.05 [95% CI (1.01–1.08); *p* = 0.006]. Regarding polygraphic parameters, increases in ODI by 1 increased the risk of insulin resistance by 3% {OR 1.03 [95% CI (1.003–1.05)]}, and increases in the obstructive AHI were associated with increased risk of insulin resistance by 2% {OR 1.02 [95% CI (1.003–1.04)]}, while increased average SpO_2_ of 1% reduced this risk by 12% {OR 0.88 [95% CI (0.78–0.98)]; *p* = 0.03}.

**TABLE 3:** Variables associated with presence of insulin resistance after 7 years.

	OR	95% IC		*P* value
**Clinical characteristics**				
Male gender	1.29	0.81	2.05	0.28
Age (years)	1.02	0.77	1.35	0.92
Weight (kg)	**1.04**	**1.02**	**1.06**	**<0**.**0001**
Obesity (BMI ≥ 30 kg/m^2^)	**3.13**	**1.58**	**6.22**	**<0**.**001**
Hip circumference (cm)	**1.03**	**1.003**	**1.06**	**0.003**
Waist circumference (cm)	**1.05**	**1.03**	**1.08**	**<0**.**0001**
Epworth scale score (unit)	1.06	0.99	1.13	0.09
Antihypertensive (Yes)	**1.72**	**1.07**	**2.78**	**0.03**
Biological characteristics				
Prediabetes state (Yes/No)	1.38	0.68	2.83	0.37
Total cholesterol (g/L)	0.62	0.33	1.17	0.14
LDLc (g/L)	0.57	0.27	1.21	0.14
HDLc (g/L)	0.66	0.36	1.24	0.2
Triglycerides (g/L)	**1.83**	**1.3**	**2.56**	**0.0005**
CRP (mg/L)	1.03	0.99	1.07	0.14
**Ambulatory blood pressure**				
24-h Systolic BP (mmHg)	1.01	0.99	1.03	0.31
24-h Diastolic BP (mmHg)	0.99	0.96	1.03	0.69
**DEXA**				
Head fat mass* (%)	1.07	0.91	1.26	0.4
Trunk fat mass* (%)	**1.05**	**1.01**	**1.08**	**0.006**
Body fat mass* (%)	1.03	1	1.06	0.05
**Respiratory polygraphy**				
SpO_2_ mean (%)	**0.88**	**0.78**	**0.98**	**0.03**
SpO_2_ min (%)	1.23	0.91	1.02	0.22
ODI (/h)	**1.03**	**1.003**	**1.05**	**0.03**
ODI ≥ 15/h	1.02	1.68	2.23	0.49
AHI (/h)	**1.02**	**1.003**	**1.04**	**0.02**
AHIo (/h)	**1.04**	**1.004**	**1.05**	**0.02**
AHIc (/h)	2.21	0.98	1.1	0.25
15 ≥ AHI > 30/h	1.01	0.8	2.32	0.26
AHI ≥ 30/h	**1.32**	**1.22**	**4.02**	**0.009**
ASR (/h)	1.23	0.9	2.05	0.66
ASR ≥ 30/h	0.86	0.53	1.02	0.91

BMI, body mass index; HDLc, high density lipoprotrein cholesterol; LDLc, low density lipoprotein cholesterol; CRP, C-reactive protein; BP, blood pressure; SpO_2_, oxyhemoglobin saturation; ODI, oxyhemoglobin desaturation index; AHIo, Apnea plus hypopnea index (obstructive mechanism); AHIc, Apnea plus hypopnea index (central mechanism); AHI, AHIc + AHIo; ASR, autonomic sleep-related micro-arousal index. *Body fat mass was measured using dual-energy X-ray absorptiometry. All significant variables are in bold.

### Multivariate Analysis

In the first model of the multivariate analysis, obesity was the only significant parameter for predicting insulin resistance [OR at 2.84; 95% CI (1.40–5.73); *p* = 0.004; Table 4]. In the second model, by adding fat mass, no variable was significantly associated with the risk of insulin resistance. In the third model, the main determinants of insulin resistance risk were triglycerides [OR 1.61; 95% CI (1.10–2.36); *p* = 0.01] and fasting blood glucose [OR 4.69; 95% CI (1.12–192.78); *p* = 0.04].

**TABLE 4:** Multiple logistic regression analysis testing variables associated with increased risk of insulin resistance after 7 years.

	Model 1 *OR [95% CI]; p-value*	Model 2 *OR [95% CI]; p-value*	Model 3 *OR [95% CI]; p-value*
**OSA severity**			
15 ≥ AHI > 30/h	1.25 [0.72–2.16]; *p* = 0.43	1.06 [0.60–1.89]; *p* = 0.83	1.20 [0.67–2.15]; *p* = 0.54
AHI ≥ 30/h	1.86 [0.99–3.48]; *p* = 0.05	1.44 [0.74–2.82]; *p* = 0.28	1.97 [0.84–4.63]; *p* = 0.24
**Gender (Male)**	1.18 [0.72–1.93]; *p* = 0.50	1.78 [0.67–4.69]; *p* = 0.24	1.43 [0.54–3.85]; *p* = 0.47
**BMI** (≥30 kg/m^2^)	**2.84 [1.40–5.73]; *p* = 0.004**	1.85 [0.80–4.28]; *p* = 0.15	1.97 [0.84–4.63]; *p* = 0.12
**DXA**			
Trunk fat mass (%)		1.06 [0.94–1.17]; *p* = 0.38	1.01 [0.90–1.13]; *p* = 0.85
Body fat mass (%)		1.003 [0.88–1.15]; *p* = 0.96	1.03 [0.90–1.18]; *p* = 0.70
**Biology**			
Blood glucose (g/L)			**4**.**69 [1**.**12–192**.**78]; *p*** = **0**.**04**
Triglycerides (g/L)			**1**.**61 [1**.**10–2**.**36]; *p*** = **0**.**01**

*BMI, body mass index; DXA, dual-energy X-ray absorptiometry. All significant variables are in bold.*

## Discussion

In this community-based elderly cohort study population, we found a low incidence of T2DM in severe asymptomatic and untreated OSA after 7 years of follow-up, without a significant association between the presence/severity of OSA and the incidence of T2DM. However, newly diagnosed asymptomatic OSA was found to be a risk factor for the presence of insulin resistance over this 7-years period. Moderate OSA was associated with a 36% increased risk of insulin resistance and severe OSA has a 2.2-fold increased risk of insulin resistance 7 years later. After adjusting for potential confounding variables, this association was primarily dependent on obesity status, fasting blood glucose and triglycerides levels at the time of entry into the study, as well as the diagnosis of OSA.

Numerous epidemiological and clinical studies have indicated increases in frequency of T2DM with age in the general population. In France, according to the National Institute of Health and Research, the prevalence of diabetes was 5.0% in 2016. The prevalence of patients with treated T2DM is highly variable and increases by age group. An analysis of reimbursements for general health insurance in 2009 estimated the prevalence of treated diabetes at 0.4% before 45 years of age, 6.3% between 45 and 64 years, 14.2% between 65 and 74 years, and 14.8% after 75 years (American Diabetes Association, 2010). A 2006 U.S. study estimated a prevalence of 18% of diabetics between the ages of 65 and 74 with a drastic (and somewhat surprising) drop in prevalence to 5.5% among those over 75 years of age (Narayan et al., 2006). In our study, the prevalence of T2DM at the beginning of the study in 2001 in subjects aged 66.2 years old was 8.3%, and the incidence of diabetics in non-apneic subjects aged 76.4 years was 3.9%. It is likely that these figures are explained by the fact that the prevalence of T2DM has been steadily increasing for more than 20 years, and that the strict selection criteria of our cohort implied the presence of a “super healthy” population with fewer risk factors for developing T2DM except for chronological age.

Clinical or population-wide cross-sectional studies evaluating the prevalence of T2DM in OSA patients showed a significantly higher prevalence, which ranged from 5 to 30% depending of the diagnostic criteria used, and with a higher prevalence in severe OSA patients (Meslier et al., 2003; Mahmood et al., 2009; Ronksley et al., 2009; Fredheim et al., 2011). Most longitudinal studies have also shown an association between OSA and T2DM. Six such studies with follow-up periods between 2.7 and 12.8 years showed the presence of a significant association between OSA and incident T2DM after adjustment for the usual confounders (Botros et al., 2009; Marshall et al., 2009; Boyko et al., 2013; Kendzerska et al., 2014; Appleton et al., 2016; Nagayoshi et al., 2016). The prospective association of sleep-disordered breathing with incident diabetes among United States Hispanic/Latino people over 6 years of follow-up has recently been published (Li et al., 2021). In this study, mild or moderate-to-severe OSA was associated with 1.33 odds of incident diabetes (95% CI 1.05–1.67) compared with no OSA. A recent meta-analysis of nine other studies which included 64,101 participants also reported that OSA is associated with T2DM with a relative “pooled” and adjusted risk ratio of 1.35 (95% CI 1.24–1.47) (Reutrakul and Mokhlesi, 2017). However, two other population-based studies evaluating this potential association reported inconclusive results, and such findings are consistent with those obtained in the current PROOF cohort study (Reichmuth et al., 2005; Marshall et al., 2009). These discrepancies could be explained by the low case number of incident T2DM in the three negative studies, or by the fact that OSA may not be an independent risk factor for T2DM in the general population unless there is and antecedent associated metabolic or phenotypic comorbidity. In other words, OSA may be operating as the second of a two-hit process, and will therefore be void of any T2DM effects if the first hit, i.e., metabolic risk, is absent. It is also important to note that in our population there are no sex differences concerning the impact of OSA on metabolic complications. This is quite unusual and unexpected, but is probably related to the inclusion criteria of the initial cohort (PROOF study) with a low incidence of android obesity. Thus, current and previous results cannot be compared with other “clinical OSA” populations.

We should also emphasize that most cross-sectional studies showing a relationship between sleep breathing disorders and carbohydrate metabolism abnormalities have shown that these disorders are associated with decreased glucose tolerance and insulin resistance (Strohl et al., 1994; Stoohs et al., 1996; Ip et al., 2002; Iftikhar et al., 2013). Such findings have been also confirmed in murine experimental models that employed lean mice and normal non-obesogenic diets (Poroyko et al., 2016; Gozal et al., 2017; Khalyfa et al., 2018).

However, the generalization of the results of these studies has been questioned due to several methodological limitations, including the use of “highly symptomatic” clinical populations, the small sample size and insufficient control for confounding factors. Notwithstanding, the results of a large cross-sectional study of a community sample of 2,656 subjects in the Sleep Heart Health Study cohort support the concept that OSA, and especially severe OSA is independently associated with glucose intolerance (pre-diabetes) and insulin resistance after adjustment for age, gender, smoking status, BMI, and self-reported sleep duration (Punjabi et al., 2002). In a cross-sectional study of 186 subjects, Murphy et al. (2017) evaluated this notion in nondiabetic obese subjects without cardiovascular comorbidity. Apneic subjects with BMI > 35kg/m^2^ had significantly higher HOMA-IR indices than controls, but such differences disappeared when the BMI was <35 kg/m^2^.

A major strength of our study is the longitudinal design and the ability to control for most confounding factors, while restricting our cohort to a narrow and relevant age range 65–75 years old (Archontogeorgis et al., 2017). We did not find a significant difference in insulin resistance indices between OSA and controls as has been shown in cross-sectional studies, but detected a significant association between OSA and insulin resistance, which was dependent on other morphometric factors. Inclusion in our multivariate models of the parameters of body composition analyzed by DXA and other blood-based measures undoubtedly allowed for improved adjustments for confounding factors. As would be anticipated, obesity was confirmed as the main confounding factor in the analysis of insulin resistance in OSA. Measures of obesity, including BMI, waist circumference and waist-to-hip circumference, have been shown to be closely correlated with insulin resistance, and central or abdominal obesity is more predictive of insulin resistance than general obesity (Ruderman et al., 1998; Murphy et al., 2017). Our regression models indicated that insulin resistance in our PROOF-SYNAPSE cohort study was highly dependent on basal BMI, trunk/head fat mass and triglyceride levels, and such factors revealed a much more dampened role of OSA, and its associated autonomic sleep fragmentation and chronic intermittent hypoxia.

We need to remark here that specific biological parameters have emerged as independent determinants of insulin resistance and exhibit an even greater effect than obesity *per se*. Blood triglyceride levels are closely correlated with insulin resistance, and as such they may mask any effect of AHI on insulin resistance. Insulin has an antilipolytic effect, promoting the storage of triglycerides in adipocytes, and reducing the release of free fatty acids into the circulation. Therefore, it makes sense to associate insulin resistance with early abnormalities in lipid metabolism, even when blood glucose remains normal. Upstream, sympathetic hyperactivity, secondary to the micro-arousals and the intermittent hypoxia associated with OSA, increases glycogenolysis and liver neoglucogenesis; and intermittent hypoxia further promotes lipolysis that contributes to insulin resistance and worsens dysmetabolic nonalcoholic hepatopathy (Kent et al., 2014). The increase in blood triglycerides as being more of a consequence of insulin resistance than an independent predisposing factor as found in our results is still debatable. Nevertheless, eating habits and lifestyle have major sway in preventing insulin resistance, hypertriglyceridemia and their consequences.

Very few clinical studies have shown that polygraphic parameters are predictors of insulin resistance. Some of such cross-sectional studies have demonstrated that nadir oxygen saturation was an independent factor of insulin resistance. A prospective study evaluating 69 nondiabetic subjects aged 49 ± 12 years showed a negative association with average oxyhemoglobin saturation, but showed no association with EEG-derived measures of sleep fragmentation (Archontogeorgis et al., 2017). Our larger cohort of older men and women does not confirm these results.

Of note, there are other known pathophysiological models explaining how chronic intermittent hypoxia can lead to the development of insulin resistance. Intermittent hypoxia could lead to glucose intolerance and insulin resistance by promoting the release of pro-inflammatory cytokines, such as interleukin-6 and tumor necrosis factor. Nocturnal and intermittent hypoxia would induce morphological and inflammatory remodeling of visceral adipose tissue (Poulain et al., 2014), with a pro-inflammatory state of subcutaneous and visceral fat cells, and negative back-control of insulin signaling (Carreras et al., 2012; Gileles-Hillel et al., 2017). Furthermore, it is likely that intermittent hypoxia operates as a pro-senescence mechanism in adipose tissues, such that the age at which onset of OSA occurs may impose differential effects on the risk of T2DM and insulin resistance (Polonis et al., 2020). It is therefore essential to keep in mind that it is necessary to carry out further large-scale prospective studies to demonstrate these pathophysiological links in humans.

Some of the strengths and limitations of our study need to be considered. The major strength is the analysis of a large, well phenotypically-defined cohort of older adults undergoing systematic periodic clinical and paraclinical assessments with homogeneity in age range, thereby allowing for more accurate estimates of the role of OSA in relation to insulin resistance and T2DM, with no possible confusion imposed by age. The use of an indirect yet well validated method evaluating insulin resistance such as the HOMA-IR is clearly preferable to glycaemic levels alone, even though insulin clamps were not performed due to their complexity and the obvious difficulty to encompass large populations (Bonora et al., 2000; Borel et al., 2018).

A first limitation of our study is the lack of baseline knowledge of the status of patients with respect to insulin resistance. This prevents us from concluding on the incidence rate of this carbohydrate control metabolism disorder according to the presence of OSA 7 years earlier. In fact, in this cohort we only have data on fasting blood glucose levels at P1.

As another limitation of the study, we should remark that participants were recruited as part of a cross-sectional survey conducted within a homogeneous group of seniors fulfilling very stringent criteria which could introduce sampling bias. It should also be noted that the initial blood samples obtained during the study precluded evaluation of insulin resistance in this population. Furthermore, all OSA patients treated during the 7-years follow-up period were also excluded from the analysis. Therefore, we cannot rule out the possibility that our overall “healthy” or “super healthy” cohort of people may not be representative of the general population, in which case our results cannot be extrapolated to OSA patients of the same age and seen in routine clinical practice. The study is a sub-study of the PROOF cohort and the data presented were obtained from a population for which it was not possible to calculate *a priori* the number of participants required. In fact, the PROOF study’s enrollment calculation indicated that 1,000 subjects were needed to assess whether or not progressive alteration of cardiac autonomic activity increased the risk of occurrence of a cardiovascular or cerebrovascular event, atrial fibrillation, or incident heart failure. Secondly, our cohort included 65-year-old subjects who could be considered “young” seniors, excluding the application of our results to “very old” subjects. Thirdly, we used an ambulatory polygraph system for the study of sleep disorders, which obviates the opportunity to assess the structure of sleep or the relationship between respiratory events and the stages of sleep that would have allowed for a more detailed analysis. However, ambulatory polygraphy is currently considered a useful and validated tool for routine screening and diagnosis of OSA even in the elderly, even if this approach underestimates the OSA severity index, i.e., AHI. Finally, OSA patients deemed “symptomatic” by their treating physician were treated and excluded from our study. Only “asymptomatic OSA” subjects remained in the study. Accordingly, it is possible that those OSA patients suffering from daytime hypersomnia may have more severe and more frequent incident cardiometabolic disorders than the asymptomatic ones, similar to the findings regarding systemic hypertension.

## Conclusion

The present study shows no evidence supporting a significant impact of asymptomatic OSA (even when severe) on the type 2 diabetes rate over a 7-years follow-up period among 66-years old people. In contrast, prediabetes or android obesity are significant risk factors associated with increased incidence of T2DM in this population. OSA, and its hallmark perturbations of chronic intermittent hypoxia and autonomic sleep fragmentation were associated with the presence of HOMA-IR at 7 years in this same cohort, even if such effect was modest and apparent only among severe asymptomatic OSA subjects. The clinical significance of these findings is elusive and improved understanding of whether “cardiometabolic control” remains an important element in the decision to treat OSA in asymptomatic elderly persons will need to be explored in future studies with the ultimate goal of reducing cardiovascular and cerebrovascular risk in this population.

## Data Availability Statement

The original contributions presented in the study are included in the article/supplementary material, further inquiries can be directed to the corresponding author.

## Ethics Statement

The studies involving human participants were reviewed and approved by CPP Sud Est 1. The patients/participants provided their written informed consent to participate in this study.

## Author Contributions

LV, J-CB, and FR: conceptualization. DH, TT, and AG: methodology. VP: software. FR, TT, and LV: validation. LV and SC: formal analysis and data curation. FR and J-CB: investigation and funding acquisition. AG: resources and project administration. LV: writing – original draft preparation. LV, FR, IC-F, and DG: writing – review and editing. FR and IC-F: supervision. All authors contributed to the article and approved the submitted version.

## Conflict of Interest

The authors declare that the research was conducted in the absence of any commercial or financial relationships that could be construed as a potential conflict of interest.

## Publisher’s Note

All claims expressed in this article are solely those of the authors and do not necessarily represent those of their affiliated organizations, or those of the publisher, the editors and the reviewers. Any product that may be evaluated in this article, or claim that may be made by its manufacturer, is not guaranteed or endorsed by the publisher.
